# Editorial: The underlying pathophysiology and therapeutic approaches for cutaneous T-cell lymphoma

**DOI:** 10.3389/fimmu.2024.1383024

**Published:** 2024-02-26

**Authors:** Patrizia Fuschiotti

**Affiliations:** Department of Medicine, Division of Rheumatology and Clinical Immunology, University of Pittsburgh School of Medicine, Pittsburgh, PA, United States

**Keywords:** cancer, skin, transcriptional, single-cellRNAseq, therapy

Cutaneous T-cell lymphomas (CTCL) are rare clonal disorders of skin-homing memory T cells. CTCL subtypes are characterized by distinct clinical and histological features. The most common variants are Mycosis fungoides (MF), where malignant T cells reside primarily in the skin, and the leukemic variant Sezary syndrome (SS). Although many T lymphocytes infiltrate the MF skin tumors and are found in the blood of SS patients, distinguishing malignant from benign cells has been impaired by a lack of specific markers for identifying and isolating the malignant cells, thus delaying diagnosis and the development of specific treatments, which results in poor clinical outcomes.

Articles and reviews included in this Research Topic report on innovative technologies that have been applied to develop newer diagnostic tools, improve prognosis, and better understand pathogenesis. Other topics of the Research Topic focus on the role of the microbiome in the pathogenesis of CTCL as well as on current skin-related or systemic therapeutic options in advanced- and early-stage CTCL. Mandel et al. report on advances in immunosequencing such as high-throughput sequencing of the T-cell receptor (HTS-TCR), which enables identification and quantification of the precise genetic signature of dominant T-cell clones. While healthy individuals present an oligoclonal T-cell repertoire, CTCL patients often exhibit aberrant proliferation of malignant T-cell clones in their skin and/or blood. The authors suggest that incorporation of HTS-TCR in clinical practice offers promise for earlier diagnosis, more accurate prognosis, and disease monitoring. Lefebvre et al. focus on recently developed single-cell methods, such as droplet-based single-cell RNA sequencing (scRNAseq), which allow profiling the transcriptomes of thousands of individual cells directly from MF/SS skin/blood tumor samples. Parallel high-resolution profiling of the T cell immune repertoire identifies the expanded T cell clones from MF/SS samples. The advantage of scRNAseq compared to HTS-TCR is that not only are the expanded clonotypes identified, but their transcriptional profiles are determined, allowing malignant T cells to be distinguished from benign. Thus, advances in single-cell technologies provide new insights into MF/SS pathogenesis that may be used to develop novel diagnostic and prognostic markers as well as identify specific targets for drug development.

Several articles in this Research Topic have highlighted the importance of scRNAseq in studying the molecular mechanisms of CTCL. Integration of the clinicopathological characteristics with scRNAseq data allowed Pan Lai et al. to determine the molecular differences between anaplastic large cell lymphoma (cALCL) and CD30-positive transformed mycosis fungoides (CD30^+^ TMF), which could improve diagnostic accuracy in clinical practice. This analysis revealed unique gene expression profiles associated with cALCL and CD30^+^ TMF. Specifically, CD30^+^ TMF showed a TCF7^+^ exhausted T-cell phenotype accompanied by B-cell infiltration, while cALCL cells expressed aberrant levels of BATF3 and HLA class II genes, polarized towards a Th17 phenotype, and exhibited neutrophil infiltration. Based on these data, the authors proposed to develop a 2-gene immunohistochemistry test to differentiate between these two diseases that could be applied in the clinical setting.

Expression of the Mas-related G protein-coupled receptor X2 (MRGPRX2) by mast cell is responsible for the IgE-independent non-histaminergic itch and has been found in the lesional skin of patients with various skin disorders. As itch is an important clinical manifestation of MF, Hu et al. employed scRNAseq to determine MRGPRX2 expression in the MF tumor microenvironment (TME). The authors demonstrated increased numbers of MRGPRX2^+^ mast cells in lesional skin of MF patients, which correlated with itch and other clinical and laboratory characteristics. However, they also identified other MRGPRX2-expressing cell-types such as sensory neurons, keratinocytes, basophils, and eosinophils, whose role in itch needs requires further investigation.

Cao et al. aimed to identify tumor-specific transcription factors underlying CTCL pathology using a previously reported scRNAseq dataset. They found that the Activating transcription factor 5 (ATF5) was specifically expressed by malignant T cells and correlated with poor treatment responses in CTCL patients. Mechanistically, they showed that ATF5 promoted the survival of malignant T cells through the PI3K/AKT/mTOR pathway, and imparted resistance to endoplasmic reticulum stress-induced apoptosis.

The treatment of MF/SS, particularly in refractory and advanced-stage, is often challenging given the absence of reliably curative therapies. In their review, Stuver and Geller highlighted recent advances in skin-directed MF/SS treatment, ranging from optimization of existing topical treatments to the introduction of novel local and topical agents. Recent progress in advanced-stage disease included the approval of brentuximab vedotin (BV) and mogamulizumab for the treatment of relapsed MF/SS. Upon binding to CD30 and internalization, BV induces cell cycle arrest and apoptosis of malignant cells, whereas Mogamulizumab binds to chemokine CCR4 on tumor lymphocytes and blocks their migration and proliferation. Ongoing clinical trials explore the potential of E7777 and lacutam in the treatment of relapsed MF/SS.

Quadri et al. discussed current and under-investigation new treatments such as monoclonal antibodies, immune checkpoint inhibitors, and Janus kinus (JAK) pathway inhibitors for advanced MF/SS. Monoclonal antibodies such as CD52, CCR4, CD30, and CD25 have been exploited as therapeutic targets for MF/SS. More recently, the monoclonal antibody Dupilumb, which targets the IL4/IL13 signaling pathway, offered great potential for MF/SS therapy. However, Dupilumab exhibited opposite effects in several case report studies and was associated with disease progression in several patients, warranting further investigation to determine the underlying mechanism(s).

Immunotherapies that target immune checkpoints, such as cytotoxic T-lymphocyte-associated protein 4 (CTLA-4) and programmed cell death protein 1 (PD-1), have been beneficial in inducing a robust and prolonged anti-tumor immune response in various malignancies. Likewise, recent studies indicated that the PD-1 inhibitors pembrolizumab and nivolumab may represent a promising approach for MF/SS therapy as well. Finally, JAK inhibitors may represent valuable molecules that target key signaling pathways in malignant lymphocytes with the potential to disrupt the progression of MF/SS. However, further studies are needed as data on JAK inhibitor efficacy in maintenance and therapy of these diseases are still inconclusive.

A more comprehensive understanding of MF/SS pathophysiology remains critical for improvement in therapeutic response and patient outcomes.

## Author contributions

PF: Writing – original draft, Writing – review & editing.

